# Association Between Periodontal Disease and Cumulative Coping Challenges: A Retrospective Cross‐Sectional Exploratory Educational Study

**DOI:** 10.1002/cre2.70457

**Published:** 2026-09-19

**Authors:** David C. Johnsen, Lauren A. G. Erickson, Derek Smith, Carlos Garaicoa‐Pazmino

**Affiliations:** ^1^ Department of Pediatric Dentistry University of Iowa College of Dentistry Iowa City Iowa USA; ^2^ Pre‐doctoral student University of Iowa College of Dentistry Iowa City Iowa USA; ^3^ Division of Biostatistics and Computational Biology University of Iowa College of Dentistry Iowa City Iowa USA; ^4^ Department of Periodontics University of Iowa, College of Dentistry Iowa City Iowa USA; ^5^ School of Dentistry Universidad de Especialidades Espíritu Santo Samborondon Ecuador

**Keywords:** coping challenges, critical thinking, periodontitis, risk assessment

## Abstract

**Background:**

Scarce scientific evidence is available on the association of cumulative coping challenges and periodontitis. The present retrospective cross‐sectional exploratory study explored a new method to depict the association between periodontal disease (extent/severity/rate of progression) and cumulative coping challenges.

**Methods:**

A population sample was identified with an array of periodontal disease levels and coping challenges within an academic setting. In January 2026, a total of 39 student‐led patient presentations were recorded using an ordinal scale based on the 2017 American Academy of Periodontology/European Federation of Periodontology framework of staging/grading and a cumulative coping challenges score, including biologic, sociologic, behavioral, demographic, and oral disability‐related factors. Spearman's correlation analysis was used to correlate periodontal disease level and cumulative coping challenges.

**Results:**

A distinct and significant association was found between level of periodontal disease and cumulative coping challenges (Spearman's *ρ *= 0.77, 95% CI, *p* < 0.01). Clusters of patients depicted that those with mild forms of periodontal disease (Stages I–II) had few coping challenges and another group with severe forms of periodontal disease (Stages III–IV) harbored higher cumulative coping challenges. No person appeared with high disease and low cumulative coping challenges, and only one person appeared with low disease and high coping challenges.

**Conclusions:**

This retrospective cross‐sectional exploratory study quantified risk factors from a series of cumulative coping challenges resulting in a single metric to be correlated with disease level based on the current staging/grading system for periodontal diseases. Periodontitis was observed among individuals with minimal disease and few coping challenges, and another group with severe stages and extensive coping challenges. Despite the theoretical limitation of the proposed scale, the present model has a practical use in an educational setting as an exploratory composite index.

## 1 Introduction

Periodontitis is a chronic multifactorial inflammatory disease associated with oral dysbiosis within a susceptible host and characterized by the progressive destruction of the tooth‐supporting structures (Papapanou et al. 2018). These dysbiotic microbial communities of keystone pathogens and pathobionts are thought to exhibit a synergistic virulence by exploiting tissue‐destructive inflammation leading to oral and systemic complications (Hajishengallis 2014). Extensive literature exists exploring on the associations of periodontal disease with a myriad of conditions related to systemic factors (e.g., diabetes, obesity, cardiovascular diseases, stress) (Genco et al. 1999; Saito et al. 2001; Sanz et al. 2018; Sanz et al. 2020), environmental habits (e.g., smoking) (Zambon et al. 1996), sociologic factors (e.g., compliance to dental care, physical disabilities) (Kotronia et al. 2019; Ravidà et al. 2021), demographic factors (e.g., financial limitations) (Chen et al. 2026), among others.

Most therapeutic efforts of periodontal therapy are focused mainly on addressing periodontal disease by means of patient education, management of periodontal‐systemic interrelationships, mechanical instrumentation, and chemotherapeutics (American Academy of Periodontology 2011). Despite our current understanding of periodontitis and technological advances, the prevalence of this condition remains high and affects an estimated 42% of dentate adults (> 30 years), with 7.8% having a severe form of periodontitis (Eke et al. 2018). The greatest risk for having any type of periodontitis was seen in older individuals, males, smokers, minority race/ethnic groups, low‐income and less‐educated groups (Eke et al. 2020).

Risk assessment is a pivotal step for all healthcare providers and essential for periodontal therapy. Assigning an individual risk level for disease progression, as part of the diagnostic process, would enable providers to determine the frequency and extent of professional support to prevent disease, achieve, and/or maintain health. Differences exist on the scale of risk values based on specialty training, as most periodontists assign risk values almost exclusively to clinical and radiographic measurements limited to periodontal disease severity (Rutger Persson et al. 2003). No single risk factor should play a paramount role above others, but instead, a multilevel risk profile should be considered when establishing a personalized risk assessment. In an early attempt, Lang and Tonetti developed the periodontal risk assessment (PRA) tool, a functional diagram for patients enrolled in periodontal maintenance, allowing customization of the recall program (Lang and Tonetti 2003).

Nonetheless, dental treatment does not always translate into achieving health among patients with recurrent disease (Johnsen et al. 2025). Some literature (Chen et al. 2026; Kotronia et al. 2019; Ravidà et al. 2021) exists on the association of periodontitis with individual upstream social factors and coping strategies/behaviors, possibly influencing the lack of response to therapy. The correlation between coping challenges and periodontitis should extend risk analysis beyond pathologic and microbiologic mechanisms. The search for a method to combine pathologic/microbiologic with behavioral/sociologic/oral disability challenges appears as the next logical step in periodontitis risk analysis.

The two areas for exploration were the concept of disability more than prevalence and the concept of cumulative coping challenges. While the current classification of periodontal diseases is not primarily intended to show disability, this classification does reflect increased disease severity with a staging system of I to IV and includes missing teeth—an indicator of disability—as well as the extent of periodontal disease (Tonetti et al. 2018). Furthermore, it is established and serves as a start toward a scale of disability until such a scale is developed. References to health and disability are seen in overall general health (Lee et al. 2022), but not to periodontal disease.

To the knowledge of the authors, we are not aware of scientific evidence establishing an altogether cumulative association of coping challenges (e.g., biological, environmental, sociological, demographic, and social risk factors) and periodontitis. In addition, no two‐dimensional exploration seems to be available for the concept of analyzing periodontal disease extent and its correlation with cumulative coping challenges. Thus, the present exploratory approach evaluates methods to depict the association between the level/extent of disease and cumulative coping challenges within an academic setting.

## 2 Methods

This retrospective cross‐sectional educational study did not meet the definition of human subjects research; thus, it was declared and granted an “Exempt status” by the University of Iowa Institutional Review Board (IRB #202601454). The requirement for informed consent was waived, and reporting followed the STROBE recommendations for cross‐sectional studies (Supporting Information S1: Appendix A).

### 2.1 Academic Setting

The academic setting was previously reported in detail elsewhere (Johnsen et al. 2025). In summary, third‐year dental students undergoing a 20‐week clerkship within the Department of Periodontics were offered a PowerPoint template to craft a 15‐min presentation based on a patient selected by the student to demonstrate critical thinking and judgment. The case presentations took place in January 2026.

Students were encouraged to select a challenging periodontal patient to hone their skills for practice. Eligible cases for inclusion were patients who had a new learning experience related to periodontics during their clerkship. These patients were selected retrospectively from the pre‐doctoral periodontics clinic and/or patients who underwent surgical procedures performed by a periodontal resident. All available data and entries were supervised by calibrated Board Certified Periodontists during patient care activities. During the selection strategy, patients were conveniently selected by the student, and consequently, these cases are not a consecutive or representative clinical sample of the general periodontal population.

Each student had a standard PowerPoint template based on a learning guide, including identical headings and subheadings. Content for this learning guide was developed over the years and came from the analysis of periodontitis, caries, geriatrics, social work, and interprofessional practice (Craig et al. 2020; Craig et al. 2022; Johnsen et al. 2025; Marchini et al. 2017). The latest version of this learning guide focuses on analyzing the disease and the person (Johnsen et al. 2025).

The student marked entries (e.g., periodontal diagnosis, prognosis, coping challenges, etc.) within the learning guide to report on the application of content relative to the student's patient. During the presentation, the faculty make entries relative to the student performance by stating if the student objectively applied the requested step and/or grasped the concept of the step. Student recordings were not changed during or after the student presentation.

Students were divided into 4 subgroups that consisted of up to 10 students per subgroup. Within each subgroup, students witnessed the presentation and patient analysis from their fellow classmates. In case of missing data (e.g., periodontal diagnosis) during the student presentation, which significantly impacted their grade, the data from this presentation was excluded.

### 2.2 Data Management for the Assessment of Periodontitis and Cumulative Coping Challenges

Once a student made an entry, the faculty recorded the student's response; thus, the faculty recording is objective based on the student's entry. Only after all students presented their patients, student responses were analyzed using the Health‐to‐Disability Scale (HDS), a two‐dimensional approach correlating the periodontal disease level with cumulative coping challenge factors (e.g., biologic, environmental, sociologic, behavioral, demographic, and oral disability challenges) (Table 1). The ideas occurred as student lamented the difficulties in interacting with patients having severe (e.g., Stage III–IV) periodontitis. The unit of analysis is the patient case presentation, not the student per se.

**Table 1:** The Health‐to‐Disability Scale (HDS), a two‐dimensional approach for the periodontitis risk assessment.

Health‐to‐Disability Scale		
Periodontal disease L	Cumulative Coping Challenges Score	
1 = Non‐periodontitis 2 = Localized/Stage I 3 = Stage II/Grade A–C 4 = Stage III/Grade A–B 5 = Stage III‐IV/Grade C	*Biologic challenges*	0/1 = Diabetes
	*Environmental challenges*	0/1 = Smoking
	*Sociologic challenges*	0/1 = Domestic stability 0/1 = Prolonged lapse in oral care 0/1 = Physical disability due to overall medical condition 0/1 = Language barrier
	*Behavioral challenges*	0/1 = Compliance
	*Demographic challenges*	0/1 = Finances 0/1 = Transportation
	*Oral disability challenges*	0/1 = Number of missing teeth (> 4) affecting quality of life 0/1 = Clinician's view/prognosis of disease progression

*Note:* The ordinal scale scores the level of periodontitis based on the 2017 AAP/EPF framework for staging/grading of periodontal diseases and a complete list of potential coping challenges in a person contributing to the severity of their periodontal condition. Coping challenges were each scored as 0 (meaning the factor is not likely to add to the risk for periodontitis) and as a 1 (meaning the factor could be a risk for periodontal disease). The cumulative challenges score is derived from the total score of assessing coping challenges. The weights of respective factors are not necessarily equal to one another.

#### 2.2.1 Assessment of Periodontitis Using the Health‐to‐Disability Scale

An ordinal scale of five scores was developed based on the 2017 AAP/EFP World Workshop of Periodontal and Peri‐implant Diseases to determine the extension, severity, and rate of disease progression of periodontitis (Tonetti et al. 2018). This proposed 5‐point disease score collapses staging and grading as follows: Non‐Periodontitis patient, Localized/Stage I (CAL: 1–2 mm, < 30% of teeth affect) Periodontitis, Generalized/Stage II Periodontitis (CAL: 3–4 mm, ≥ 30% teeth affected), Stage III/Grade A‐B Periodontitis (CAL ≥ 5 mm with minimal loss of dentition) and Stage III–IV/Grade C Periodontitis (CAL ≥ 5 mm with minimal/extensive loss of dentition). Although this categorization is not validated, this scale mix extension, severity, and rate of progression of periodontal disease in a way that may not be linear. A misclassification risk can happen when students rely on clinic diagnoses that may vary between providers. For this purpose, data were taken directly from the presentation to reduce the risk for bias.

The diagnosis of the extension, severity, and disease progression was determined during patient care and prior to the presentation with the main provider and clinical instructor making the determination. The student used that determination for their presentation, and the faculty in the seminar did not change the disease level/extent determination made in the clinic.

#### 2.2.2 Assessment of Cumulative Coping Challenges Using the Health‐to‐Disability Scale

To explore cumulative coping challenges, categories were selected that were on the student's learning guide and considered to have a potential association with disease (e.g., periodontitis and/or caries). Unlike the scale for periodontal disease, no attempt was made to develop a scale from lowest to highest for cumulative coping challenges. The retrospective hypothesis is that the cumulative risk factors for periodontitis may have a correlation with the extension/severity/rate of progression of the disease.

Similarly, no attempt was made to equalize either the different potential coping challenges or the different domains of the burdens. The selected coping challenges were from different domains, including biological, sociological, behavioral, demographic, and oral disability. Factors directly affecting the patient's biology were conditions such as smoking and diabetes mellitus. Sociological coping challenges included domestic stability, prolonged lapse in oral care, physical disability, overall medical condition, and language barrier. Compliance was the main behavioral coping challenge. Among demographic challenges were finances and transportation (e.g., total travel distance/time to the College of Dentistry). Lastly, oral disability coping challenges were associated with multiple missing teeth (≥ 5 teeth lost due to periodontitis affecting quality of life) and the clinician's view/prognosis of disease progression.

We were not aware of attempts to combine coping challenges from different domains into one analysis. The categories for coping challenges were not considered equivalent. Nonetheless, the notion seemed logical that more than one challenge can accumulate in affecting the disease level. Each challenge was scored as a “1” if considered a potential associate with disease and a “0” if not. The total number for the score “1” made up the score for an individual patient. Thus, this exploratory project assumes identical weight for highly heterogeneous constructs (e.g., controlled diabetes vs. language barrier vs. 5 missing teeth), and several variables (e.g., clinician's view of prognosis) are inherently subjective.

Coping challenges related to transportation, lapse in oral care, and missing teeth, qualifications were made after the presentation. For transportation, the need for assistance in driving and a driving distance over 1 h were designated as a “1”. The lapse in oral care extending for multiple years (> 5 years) was designated as a “1”. For multiple missing teeth, four or more missing teeth (other than third molars or orthodontic extractions) were designated as a “1”. Specific potential coping challenges were selected individually from the template the students used and were not added later. The score was calculated as an unweighted sum of binary variables, although the different domains contribute unequal numbers of items, resulting in unequal weighting across domains. Additionally, this index is presented as an exploratory composite measure rather than a validated scale unless psychometric properties are provided.

### 2.3 Statistical Analysis

A simulation study based on the available sample size and assumed score distribution revealed that a Jonckheere‐Terpstra test had an approximately 80% power to detect a monotonic trend equivalent to a proportional‐odds log‐odds increase of approximately 0.5 per disease category. Spearman correlation analysis and a Jonckheere‐Terpstra test were used to assess the correlation between the 5‐level periodontitis score and the cumulative coping challenges score. The 95% confidence interval for rho was computed using Fisher's *z* transformation with the standard error of Bonett and Wright. All statistical analyses were performed with R^1^. The type I error threshold was set to 0.05.

## 3 Results

Data were obtained from the faculty assessment instrument (replica of the learning guide) and PowerPoint presentations with extensive details from a total of 39 out of 40 dental students, serving as the basis of the current project. Data from one case presentation was excluded as not all the variables were available for proper analysis.

### 3.1 Assessment of Periodontal Disease Based on HDS

Using the HDS scale, an array of scores of periodontitis was found from the student presentations. Among 39 patients, 13 patients (33.33%) were designated as Score 1 (Non‐periodontitis); 10 patients (25.64%) were designated as Score 2 (Localized/Stage I); 3 patients (7.69%) were designated as Score 3 (Generalized/Stage II); 8 patients (20.51%) were designated as Score 4 (Generalized/Stage III/Grade A‐B); and ultimately, five patients (12.82%) were designated as Score 5 (Generalized/Stage III–IV/Grade C).

### 3.2 Assessment of Coping Challenge Based on HDS

Based on all 39 patients, 10 patients (25.64%) were designated to be smokers; 5 patients (12.82%) were designated as diabetics (controlled/non‐controlled); 12 patients (30.76%) were designated for domestic instability; 11 patients (28.20%) were designated to have financial limitations; 16 patients (41.02%) were designated for possess transportation issues; 20 patients (51.28%) were designated to display signs of non‐compliance; 15 patients (38.46%) were designated to have unfavorable prognosis based on the clinician's view of periodontal disease progression; 9 patients (23.07%) were designated to have a prolonged lapse in oral care; 11 patients (28.20%) were designated to have multiple (> 4 teeth) missing teeth lost due to periodontal disease; and 14 patients (35.89%) were designated as having a physical disability, limiting medical condition or language barrier.

### 3.3 Correlation Between the Periodontal Disease Level and Cumulative Coping Challenges

**Figure 1:**
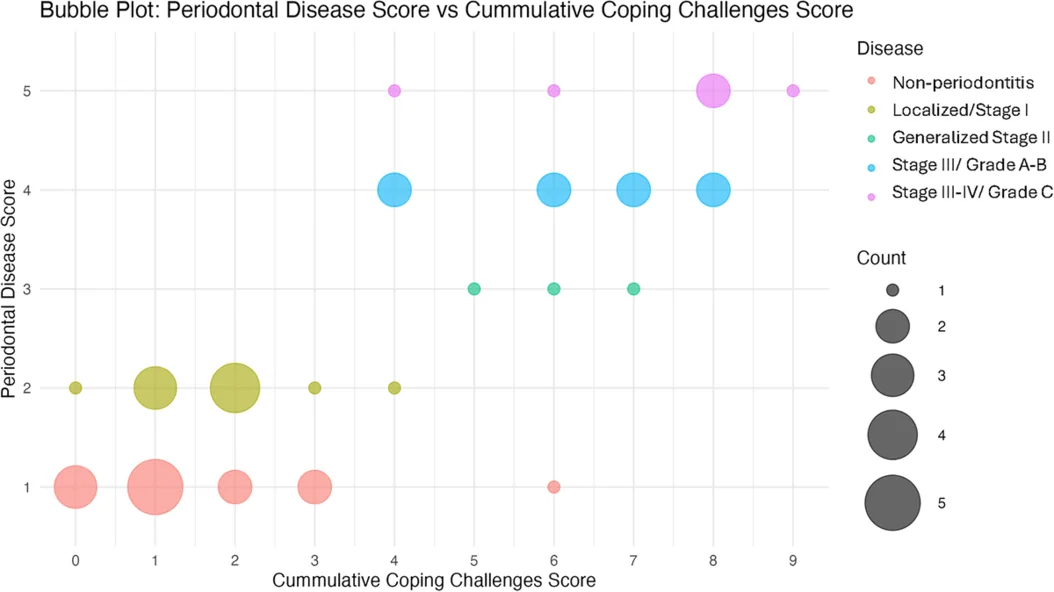
Correlation between periodontal disease and cumulative coping challenges scores. The severity of periodontitis is depicted on the vertical axis and the cumulative coping challenges for the patient on the horizontal axis, showing a strong positive correlation (Spearman's *ρ* = 0.77; *p*‐value = < 0.001). Each number represents one patient included in each of the presentations (*n* = 39). The association between the extent of periodontal disease and the cumulative coping challenges score was assessed using Spearman's rank correlation. The 95% confidence interval for rho was computed using Fisher's *z* transformation with the standard error of Bonett and Wright.

Data for individual patients correlating periodontal disease extent and cumulative coping challenges depicted a strong positive correlation (Spearman's *ρ* = 0.77; *p*‐value = < 0.001) (Figure 1). For patients with periodontal disease scores of 1 or 2, 20 patients (51.28%) had a cumulative coping challenges score of ≤ 3, and only three patients (7.69%) had a cumulative coping challenges score of > 3. Interestingly, none of the patients with periodontal disease scores of 3–5 had a cumulative coping score of < 4, whereas 16 patients (41.02%) had a cumulative coping score of ≥ 4, showing a positive correlation between the severity and extension of periodontitis and coping challenges. Only two patients (5.12%) appeared as potential outliers with periodontitis scores of 1‐2 and a cumulative coping challenge score of ≥ 4. Patients with more extensive periodontal disease had significantly higher cumulative coping challenges scores, with the score rising consistently from category 1 through category 5 (Jonckheere–Terpstra test, *z* = 5.05, *p* < 0.001) (Table 2).

**Table 2:** Cumulative coping challenge score by extent of periodontal disease.

Disease category	Sample size (*n* = 39)	Median coping score	IQR (Q1–Q3)
1	13	1	1–2
2	10	2	1–2
3	3	6	5.5–6.5
4	8	6.5	5.5–7.25
5	5	8	6–8

*Note:* Coping challenges score is the sum of 11 binary items (Range 0–9). Quartiles calculated using the default (Type 7) definition.

A further analysis was done for a cluster of individuals comparing low/high periodontal disease scores and low/high coping challenges. For each person, an abbreviated notation of the intervention completed or planned is presented in Table 3. Only the final treatment plan or actual intervention was noted in the table and clearly demonstrated a distinctly different type of procedures. Procedures with lower scores of periodontitis are meant to improve the person's oral health. On the contrary, interventions to arrest disease progression are observed among higher scores of periodontitis.

**Table 3:** Interventions rendered among the selected patients with low versus high levels of periodontal disease and coping challenges.

Low levels of PD/Low Coping Challenges		High levels of PD/High Coping Challenges	
Person #	Intervention	Person #	Intervention
1	Esthetic Crown Lengthening	2	SRP and extraction of periodontally hopeless teeth
10	Esthetic Crown Lengthening	9	Extraction of periodontally hopeless teeth
13	Connective tissue graft	11	SRP and periodontal abscess
20	SRP and Periodontal Re‐evaluation	14	SRP and Periodontal Re‐evaluation
22	Free gingival graft	24	SRP and Smoking cessation
33	Free gingival graft	25	SRP and extraction of periodontally hopeless teeth
36	Implant therapy	27	SRP and Periodontal Maintenance
37	Extraction and Implant therapy	28	SRP and extraction of periodontally hopeless teeth
		29	SRP and Periodontal Re‐evaluation
		39	SRP and Smoking Cessation

Abbreviations: PD. Periodontal disease; SRP, Scaling and root planing.

*Note:* No statistical comparison between intervention patterns was performed.

## 4 Discussion

Over the last decades, the relationship between chronic inflammatory conditions, such as periodontitis, and the occurrence of stressful life events, personality, and coping skills has been established. Despite the cumulative evidence indicating this correlation (Genco et al. 1999; Green et al. 1986; Hugoson et al. 2002), it might explain why patients with extensive and recurring disease may call for escalating interventions in an attempt to slow disease progression (Johnsen et al. 2025). A personalized two‐dimensional patient risk assessment should be a prominent theme and critical step among individuals dealing with periodontal disease.

Although available periodontal risk assessment tools (e.g., PRA) may help clinicians determining risk for disease progression including primarily clinical (e.g., bleeding on probing, pocket depths, tooth loss, radiographic bone loss), environmental (e.g., smoking) and patient/systemic variables (e.g., genetics, age, diabetes, etc.) (Lang and Tonetti 2003), the possibility of consolidating coping challenges with periodontitis orients the clinician's risk analysis to a disease‐based reference point rather than to an arbitrary reference point (e.g., low, medium, high). An early study found that periodontal patients with inadequate defense coping behaviors are at a greater risk for severe periodontal disease (Wimmer et al. 2002). A factor analysis and Varimax rotation were able to identify different factors (e.g., resigned, active, distractive, defensive, pharmaceutical/aggression) associated with inappropriate coping styles. Following treatment, patients with passive coping strategies showed a more pronounced response in cases with poor response to non‐surgical periodontal therapy (Wimmer et al. 2005). If the concept of a cumulative coping challenge to include multiple domains holds promise, this model in the present study is an early first step with much refinement to follow.

For an education framework, the realization that disease level is associated with coping challenges orients the student/clinician to sharpen the analysis for the patient's capacity to subscribe to professional recommendations. The student/clinician judgment on the patient's capacity does not change the commitment to give the patient every chance to gain sustained health, but does bring in the perspective of setting realistic goals. The present model is a compromise to consolidate an array of coping challenges into one metric against the inherent inequality of respective challenges. This model assumes that each challenge from the different domains is worthy of consideration in the overall periodontitis risk analysis. With inherent limitations on assigning a “0” or “1” to each variable, the assignment of a number assures that each coping challenge will be considered in patient planning. For example, diabetes and patient compliance are both important; however, it is beyond the scope of this project to say which is more important and by how much. Assigning a value to each variable for an overall consideration of coping challenges is unique. The use of a “0” or “1” is practical in a clinical setting, giving the model potential use in the clinical/teaching setting to increase assurance to consider each challenge cumulatively. While the endpoint of the HDS does differ notably from established tools (e.g., disease progression for the PRA tool), HDS is seen to be a step closer to a scale to measure from health to disability and the possible correlation with cumulative coping challenges.

This two‐dimensional cross‐sectional analysis between periodontal disease level and cumulative coping challenges offers novice and experienced clinicians the opportunity to gauge the challenges in crafting a treatment plan in concert with the person's compliance capacity. Nonetheless, expert clinicians may intuitively have already grasped what they are dealing with regarding escalating interventions for a given patient without the benefit of visual analysis for disease extent correlated with cumulative coping challenges. Conversely, the visually striking feature of the depiction of the level of periodontal disease versus the cumulative coping challenges is the distinct slope. With no patients displaying high disease and low cumulative coping scores, the data pattern can have an impact on novice and young clinicians. If a single risk factor dominated a patient's condition, these patients may have appeared as outliers in areas under/over the slope, thus highlighting the importance of these cumulative coping challenges during treatment planning and prognosis. Nonetheless, the potential of this cumulative coping challenge is a crude index comprised of an unweighted sum, and the reported correlation may be heavily driven by a subset of dominant factors (e.g., smoking, diabetes, tooth loss).

This exploratory retrospective model includes a population sample within an academic setting, which may suggest a clinical cohort as much as an educational sample. Given the cross‐sectional design, the findings are best interpreted as demonstrating an association rather than a predictive or causal relationship. Coping challenges are seen as different from risk factors, emphasizing instead their association with periodontal disease level, highlighting the exploratory and hypothesis‐generating nature of the proposed framework. While the educational potential is a two‐dimensional depiction for a patient, the methodology is a correlation between an ordinal disease score and a summed index, presented as Figure 1.

The association of disease extent and cumulative coping challenges is worth further discussion from the standpoint of thresholds for the patient. For example, where does disease challenge become disease burden? An assumption is that increasing accumulation of coping challenges at some point becomes burdens exceeding the patient's capacity to cope. At some point, the shift for the student/practitioner is from the mindset of disease management (which implies control) to disease mitigation (which implies damage control). The project is supportive of the notion that people with high disease burden may have challenges in bringing the disease under control and achieving sustained health. A survey of practitioners showed skepticism that a person with recurring disease can convert to sustained health (Johnsen et al. 2026). On the flip side, all respondents agreed that patients should be offered every opportunity to gain sustained health. Despite individualized and more targeted approaches being on the rise, there is a lack of prospective clinical evidence showing the cumulative contributions of specific mechanisms in the progression of periodontitis (Kornman 2018).

For future exploration is the comparison of one‐dimensional and two‐dimensional analyses for periodontitis risk in real clinical settings. The question remains whether a tolerable level of subjectivity is worth the exchange for a two‐dimensional perspective; this search would be for more objective measures common to a series of diverse factors associated with periodontitis. The present two‐dimensional model may indicate that common biologic factors (i.e., diabetes, smoking) can be combined with other risk factors (i.e., demographic, sociologic, behavioral) that are not separate in a full analysis.

One potential strength of this project is the breadth of patients by selecting patients from the combined pre‐doctoral and graduate periodontal clinics. A more homogeneous patient pool might not offer the breadth of disease level and coping challenges. On the contrary, the inherent subjectivity of several coping challenges is perhaps the biggest caution. While the selected categories have shown an association with disease, the methods to quantify some of the categories call for refinement (Genco et al. 1999; Wimmer et al. 2002).

An important limitation is sample size with student‐generated data, even though Board Certified Periodontists oversaw each patient analysis in a real clinical setting. Selective inclusion of complex cases by the student may influence the observed association and is seen as a limitation.

The theoretical limitation is the grouping of coping challenges into one metric, as the different coping challenges are not equal to one another. Several variables included in the HDS (e.g., tooth loss due to periodontitis, prognosis, prolonged lapse in care, and compliance) are conceptually related to, or consequences of, periodontal disease itself. Thus, this overlap may contribute to the observed association. Moreover, the project is exploratory in nature, and the combination of this array of coping challenges possesses some inherent limitations. There is no exploration of whether specific domains (e.g., biologic vs. sociologic vs. behavioral) drive the reported association, and no attempt is made to examine potential confounding between factors (e.g., age, tooth loss, smoking, etc.). Thus, future validation studies may consider evaluating the performance of the index using only variables that are independent of periodontal disease level.

Ultimately, this exploratory, unvalidated composite index uses quantification for practical use with limitations on robustness as an epidemiologic tool. A paradox is that the model offers a two‐dimensional view from a person's perspective and not by their pathology. The clear association of periodontal disease level and cumulative coping challenges seen by the experienced clinician does not increase the validity of the instrument.

## 5 Conclusion

This retrospective cross‐sectional exploratory study quantified risk factors from a series of cumulative coping challenges resulting in a single metric to be correlated with periodontal disease level based on the current staging/grading system. Periodontitis was observed among individuals with minimal disease and few coping challenges, and another group with severe stages and extensive coping challenges. Despite the theoretical limitations of the present scale and reported correlations, the significance for the clinician is the awareness of coping challenges to be realized in planning interventions within the patient's capacity.

## Author Contributions

**David C. Johnsen:** conceptualization (Lead), methodology (Lead), data curation (equal), formal analysis (lead), writing – original draft preparation (equal). **Lauren A.G. Erickson:** writing – original draft preparation (equal). **Derek Smith:** validation (lead), writing – review and editing (equal). **Carlos Garaicoa‐Pazmino:** writing – original draft preparation (equal), visualization (lead). All authors gave their final approval and agreed to be accountable for all aspects of the work.

## Funding

The authors have nothing to report.

## Conflicts of Interest

The authors declare no conflicts of interest.

## One‐Sentence Summary

Strong positive correlation between periodontitis and cumulative coping challenges.

## Supporting information


**Appendix A.** STROBE checklist for cross‐sectional studies.

## Data Availability

The authors have nothing to report.

## Footnotes

1 R Core Team, Vienna, Austria.
